# Anomalous Pattern of Left Hemisphere Visual Connectivity in Children With Autism: Association With Impaired Praxis

**DOI:** 10.1002/aur.70146

**Published:** 2025-11-29

**Authors:** Jonah McLaughlin, Deana Crocetti, Stewart H. Mostofsky, Daniel E. Lidstone

**Affiliations:** ^1^ Center for Neurodevelopmental and Imaging Research Kennedy Krieger Institute Baltimore Maryland United States; ^2^ Department of Neurology Johns Hopkins University School of Medicine Baltimore Maryland United States; ^3^ Department of Psychiatry & Behavioral Sciences Johns Hopkins University School of Medicine Baltimore Maryland United States; ^4^ School of Behavioral Sciences and Education Penn State Harrisburg Middletown Pennsylvania United States

## Abstract

Prominent theories of autism suggest autism‐associated differences in visual‐motor integration (VMI) may disrupt learning of motor and social skills typically acquired by observation and imitation. Supporting these theories, children with autism spectrum disorder (ASD) show robust differences in motor tasks reliant on dynamic VMI (e.g., ball‐catching and motor imitation) and anomalous visual‐motor connectivity between higher‐order visual (HOV) and sensory‐motor cortices. Use of functional MRI (fMRI) to examine HOV functional connectivity (FC) has been particularly revealing with other conditions. For instance, research with congenitally blind adults reveals a particular pattern of altered HOV connectivity, showing reduced HOV connectivity with primary sensory‐motor (SM1) and primary auditory (A1) cortices yet “compensatory” increased connectivity between HOV and prefrontal cortex (PFC). Informed by these findings, we used fMRI to examine HOV FC in children with ASD, hypothesizing they would show a distinct pattern of HOV connectivity relative to typically developing (TD) children, with decreased HOV‐SM1 connectivity and increased “compensatory” HOV‐PFC connectivity. We further hypothesized that this altered pattern of HOV connectivity would correlate with autism‐associated difficulties with performing skilled actions (“praxis”), often learned through visual imitation. Our findings suggest ASD children show an altered pattern of HOV connectivity that is characterized by reduced HOV connectivity with SM1 yet increased connectivity with PFC. Further, this HOV connectivity correlated with impaired praxis in children with ASD, suggesting that altered patterns of HOV connectivity may contribute to difficulty acquiring a range of skilled behaviors observed in autism.

**Summary**

- Evidence indicates that infants, due to their inherently minimal visual experience, exhibit a higher‐order visual (HOV) connectivity profile more similar to congenitally blind adults than to sighted adults. This suggests that visual experience itself plays a crucial role in developing visual areas.
- Children with autism spectrum disorder (ASD) show significant differences in motor tasks that rely on dynamic visual‐motor integration, potentially the result of altered visual experience during development.
- The current study observed an altered pattern of HOV connectivity characterized by reduced HOV connectivity with primary sensory‐motor cortex (SM1) and increased connectivity with prefrontal cortex (PFC). In children with ASD, this HOV connectivity correlated with impaired praxis: the ability to perform skilled actions acquired through visual imitation.

## 1 Introduction

Autism Spectrum Disorder (ASD) is a neurodevelopmental disorder characterized by impairments in social communication and restricted, repetitive patterns of behavior and interests (American Psychiatric Association 2022). Motor impairments are frequently highlighted features with a prevalence of 87% among children with ASD (Bhat 2021). Understanding these motor impairments in ASD may lead to insights into the specific neural mechanisms that underlie the disorder. Motor imitation, in particular, has received growing attention as a potential link between the motor and social communication differences characteristic of ASD. Identifying the brain mechanisms associated with differences in motor imitation may inform the development of targeted interventions to support both motor and social skill development in children with ASD.

Resting‐state functional MRI (rs‐fMRI) studies have found differences in connectivity between higher‐order visual (HOV) and motor networks that were predictive of ASD core symptoms and differences in motor imitation and praxis suggesting visual‐motor integration (VMI) may play a role (Oldehinkel et al. 2019; Nebel et al. 2016). Prominent theories of ASD suggest autism‐associated differences in VMI may disrupt the learning of motor and social skills typically acquired by observation and imitation (Mostofsky and Ewen 2011; Lidstone and Mostofsky 2021). Supporting these theories, children with ASD show robust differences in motor tasks that require dynamic tracking of visual stimuli to guide or plan motor actions (i.e., dynamic VMI) (Lidstone and Mostofsky 2021). Dynamic visual stimuli may therefore result in degrees of perceptual blindness whereby children with ASD may “miss” fast‐moving social and motor visual stimuli (Gepner and Féron 2009). As a result, they may develop compensatory brain connectivity patterns, similar to infants and individuals who are congenitally blind, to help manage difficulties integrating dynamic visual input with the motor system. Recent work by Tian et al. (2024) examining the functional connectivity (FC) profiles of infants, congenitally blind adults, and sighted adults revealed profound similarities in FC between infants, who have minimal visual experience, and congenitally blind adults. Specifically, both infants and congenitally blind adults exhibit reduced HOV connectivity with primary sensory‐motor (SM1) and primary auditory (A1) cortices yet “compensatory” increased connectivity between HOV and prefrontal cortex (PFC) relative to sighted adults. These results suggest infants require visual experience to eventually develop the FC patterns observed in sighted adults. Thus, dynamic VMI differences in children with ASD could be the product of altered visual experience over the course of development. There is reason to believe children with ASD experience vision differently relative to their TD counterparts. Individuals with ASD show a stronger preference for proprioceptive over visual input, which correlates with greater autism severity and differences in motor imitation (Glazebrook et al. 2009; Haswell et al. 2009; Izawa et al. 2012; Marko et al. 2015; Lidstone et al. 2025).

This study uses resting‐state fMRI (rs‐fMRI) to examine whether children with ASD exhibit a compensatory pattern of brain connectivity similar to that seen in infants with limited visual experience and congenitally blind individuals. We hypothesized that, compared to typically developing (TD) children, children with ASD would show reduced FC between the HOV cortex and primary sensorimotor cortex (SM1), alongside increased compensatory connectivity between HOV and PFC. Using rs‐fMRI data to assess connectivity between these regions, we expected children with ASD to show a pattern more similar to infants than their TD peers. Specifically, we predicted a distinct HOV connectivity profile characterized by decreased HOV–SM1 and increased HOV–PFC connectivity. Finally, we hypothesized that this altered HOV connectivity would correlate with autism‐related difficulties in performing skilled actions (praxis), which are often acquired through dynamic VMI.

## 2 Methods

### 2.1 Participants

The sample included 427 children, 127 ASD (27 girls) and 300 TD (98 girls) aged 8–12 years (M ± SD; males: 10.5 ± 1.31, females: 10.3 ± 1.25) that completed resting fMRI (see Table 1). Within the ASD sample, 77 children (17 girls) had comorbid ADHD. For detailed diagnostic inclusion/exclusion criteria and ASD comorbidities see Data S1.

**TABLE 1:** Demographic information with praxis and ASD core symptom breakdowns.

	TD (*N* = 300)	ASD (*N* = 127)	*p*	*N*
Age	10.29 ± 1.21 (8.02–12.98)	10.56 ± 1.35 (8.06–12.99)	0.049	427
Sex	67.3% M, 32.7% F	78.7% M, 21.26% F	0.024	427
Handedness	6.7% L, 85.7% R	9.5% L, 82.7% R	0.6	427
Head coil	47.3% 8 ch, 52.7% 32 ch	45.7% 8 ch, 54.3% 32 ch	0.835	427
IQ	116.82 ± 12.45 (87–157)	108.01 ± 16.49 (61–148)	< 0.001	427
Praxis	20.51 ± 9.42 (5.0–61.5)	39.79 ± 18.70 (9.5–84.5)	< 0.001	293
ADOS	—	11.89 ± 3.54 (6–21)	< 0.001	125
SRS	43.42 ± 5.47 (34–80)	74.73 ± 10.39 (50–90)	< 0.001	351

### 2.2 Measures and Exclusions

Resting‐state fMRI was collected on 3 T Philips scanners with eight or 32 channel head coils at the F.M. Kirby Research Center for Functional Brain Imaging at the Kennedy Krieger Institute. Autism Diagnostic Observation Schedule (ADOS) and Social Responsiveness Scale (SRS) were included as measures of ASD core symptom severity. Wechsler Intelligence Scale for Children (WISC) General Ability Index (GAI) was used as a measure of IQ. Edinburgh Handedness Index was used to determine the handedness of participants; Physical and Neurological Examination for Subtle Signs (PANESS) preferential hand was used instead for cases where Edinburgh was not acquired. Apraxia was measured using a version of the Florida Apraxia Battery modified for children (Mostofsky et al. 2006) and the primary outcome was average total errors with greater errors corresponding with worse praxis.

Participants with rs‐fMRI data with less than 5 min (TR 2.5) of continuous low motion data were excluded from the analytic sample; low motion was defined as having less than 3 mm of translation and 3° of rotation. Children with current psychotropic medication use and/or a history of intellectual disability, seizures, traumatic brain injuries, neurodevelopmental or psychiatric disorders, or other neurological illnesses were excluded from the study. Children with a full‐scale IQ (FSIQ) less than 60 on the WISC were also excluded. Praxis scores were only obtained from 293 children due to time constraints during study visits.

### 2.3 Data Processing and Analysis

**FIGURE 1:**
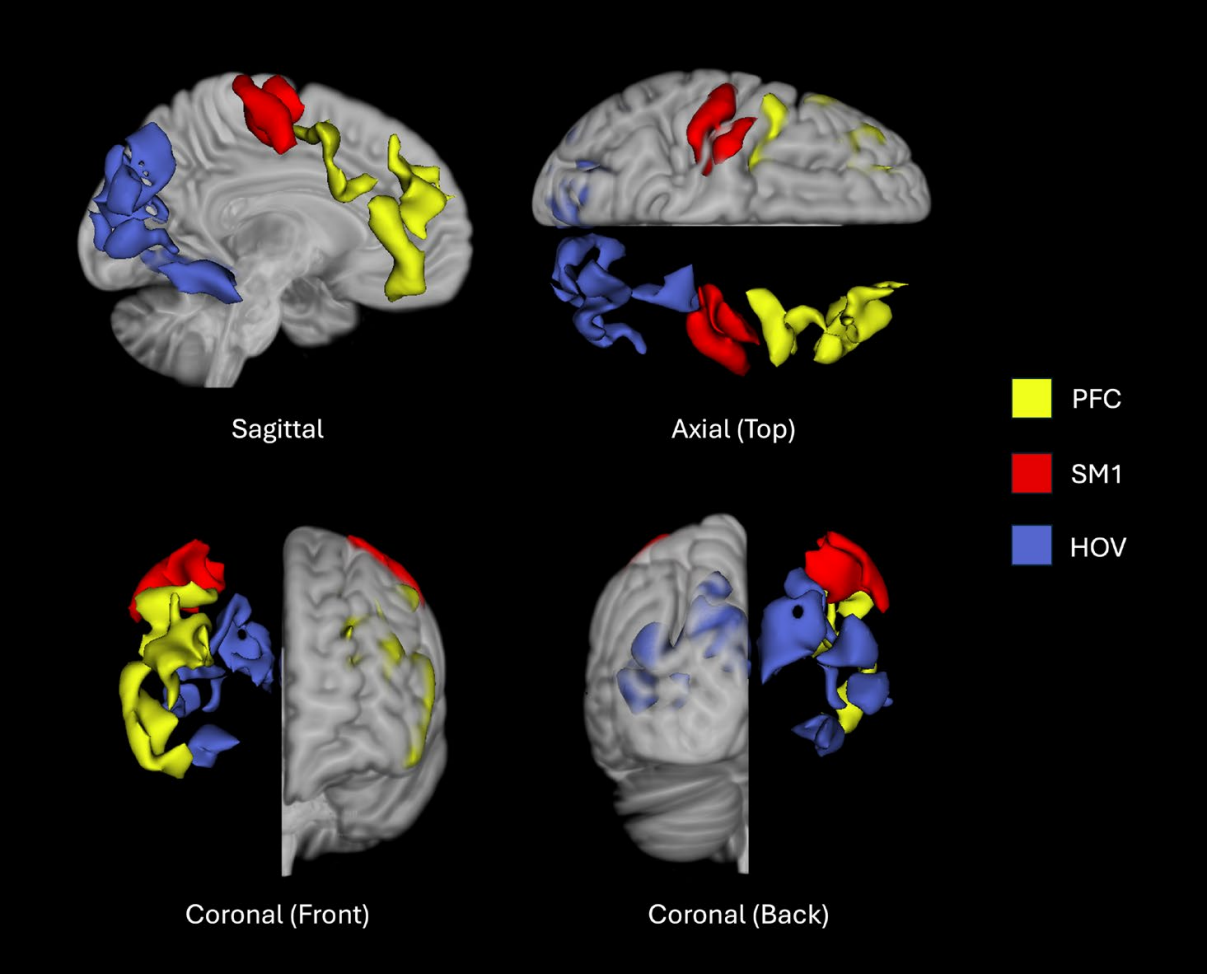
Regions of interests (ROIs) from Tian et al. (2024) with PFC ROIs in yellow, SM1 in red, and HOV in blue.

Seed‐based analyses were performed on resting‐state fMRI data using an in‐house pipeline, which included nuisance regression (aCompCor). Interregional FC was assessed between HOV, SM1, and PFC ROIs as defined in a previous study (Tian et al. 2024; see Figure 1). Multiple linear mixed‐effects models were employed to examine diagnosis‐by‐ROI interactions. Additionally, separate multiple linear mixed‐effect models were applied to each ROI to examine diagnosis and diagnosis‐by‐age interaction effects using R version 4.2.2. Model covariates included handedness, IQ (GAI), sex, and head coil. Follow‐up post hoc analysis was performed using the *emmeans* function with FDR multiple comparisons correction.

## 3 Results

**FIGURE 2:**
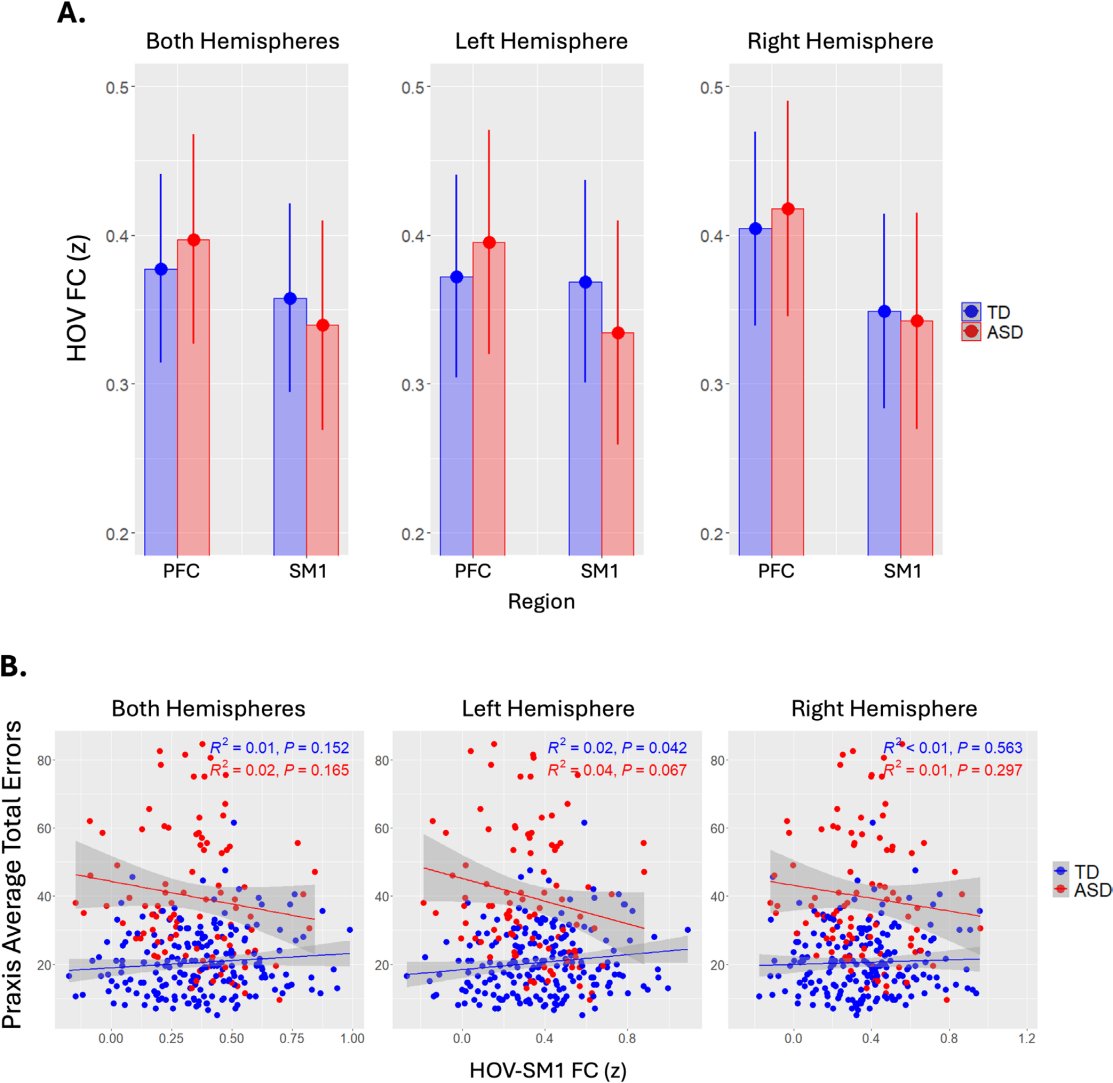
(A) HOV‐PFC FC versus HOVA‐SM1 FC (z) split by Dx across hemispheres. (B) raw praxis average total errors plotted against HOV‐SM1 FC (z) across hemispheres.

A significant diagnosis‐by‐ROI interaction was observed between HOV‐PFC and HOV‐SM1 connectivity (p=0.04). Compared to TD children, those with ASD displayed reduced HOV‐SM1 connectivity and greater HOV‐PFC connectivity (see Figure 2A). Examining left and right hemispheres independently, revealed significance in left hemisphere (p=0.0087) and not right hemisphere (p=0.33). Left hemisphere post hoc comparisons indicate mean HOV‐PFC FC (M=0.39, SE=0.022) was significantly greater than mean HOV‐SM1 FC (M=0.33, SE=0.022) in those with ASD (p=0.006) and not TD (p=0.77) after FDR multiple comparisons correction (see Table 2). Right hemisphere post hoc comparisons indicate mean HOV‐PFC FC (M=0.43,SE=0.26 in ASD; M=0.41,SE=0.02 in TD), where M represents the estimated marginal mean, was significantly greater than mean HOV‐SM1 FC (M=0.35,SE=0.026 in ASD; M=0.36,SE=0.02 in TD) in both diagnostic groups (see Table 3).

**TABLE 2:** Left hemisphere post hoc differences across diagnosis and left HOV FC ROI levels. *p* values are FDR corrected.

Left hemisphere	Mean difference	SE	*t*	*p*
TD PFC—ASD PFC	−0.015	0.025	−0.61	0.65
TD PFC—TD SM1	0.0035	0.012	0.29	0.77
TD PFC—ASD SM1	0.046	0.025	1.85	0.17
ASD PFC—TD SM1	0.019	0.025	0.75	0.65
ASD PFC—ASD SM1	0.061	0.018	3.34	0.0056^*^
TD SM1—ASD SM1	0.042	0.025	1.71	0.17

* *p* < 0.05.

**TABLE 3:** Right hemisphere post hoc differences across diagnosis and right HOV FC ROI levels. *p* values are FDR corrected.

Right hemisphere	Mean difference	SE	*t*	*p*
TD PFC—ASD PFC	−0.014	0.025	−0.55	0.70
TD PFC—TD SM1	0.055	0.012	4.81	< 0.0001^*^
TD PFC—ASD SM1	0.062	0.025	2.50	0.019^*^
ASD PFC—TD SM1	0.069	0.025	2.78	0.011^*^
ASD PFC—ASD SM1	0.076	0.018	4.29	0.0001^*^
TD SM1—ASD SM1	0.069	0.025	0.28	0.78

* *p* < 0.05.

No significant diagnosis‐by‐age effects were observed. Associations with praxis revealed a significant diagnosis‐by‐HOV‐SM1 connectivity interaction for praxis average total errors in left hemisphere (< 0.0001), where increased HOV‐SM1 connectivity was associated with better praxis for ASD, but worse praxis for TD; overall model $R^{2}$= 0.496, < 0.0001. No significant associations were observed between core autism symptoms (ADOS‐2 Total, SRS‐2 Total) and HOV connectivity.

## 4 Discussion

Our findings show a distinct pattern of left hemisphere HOV connectivity in ASD children compared to TD children. This pattern of connectivity was characterized by reduced connectivity between HOV and SM1 regions, along with increased connectivity between HOV and PFC. Further, decreased connectivity between HOV and SM1 in the left hemisphere strongly predicts worse praxis in ASD children, but better praxis in TD children—a finding that was not observed for the right hemisphere.

The observed left‐hemisphere pattern of decreased HOV‐SM1 connectivity and increased HOV‐PFC connectivity in ASD aligns with a compensatory or immature connectivity profile that has been observed in infants, who have minimal visual experience, and congenitally blind adults (Tian et al. 2024). The increased HOV‐PFC connectivity likely reflects greater reliance on cognitive control and executive processes to compensate for reduced integration of visual input with the motor system (i.e., VMI), particularly in the left hemisphere. This interpretation aligns with theories proposing that differences in VMI contribute to disruptions in learning motor and social skills often learned through observation and imitation (Mostofsky and Ewen 2011; Lidstone and Mostofsky 2021). It is possible that the observed differences in connectivity patterns and their associated disruption to praxis stem from more fundamental deficits in multisensory integration, which may contribute to difficulties coordinating the integration of visual and motor information when performing skilled actions (Wymbs et al. 2021).

The specific left‐hemispheric nature of these findings is particularly noteworthy. We observed left hemispheric HOV‐SM1 connectivity strongly predicts the ability to perform skilled actions (praxis), even more so than right hemisphere HOV‐SM1 connectivity. This agrees with previous research demonstrating left‐lateralized inferior parietal activation during motor imitation and findings that left hemisphere stroke patients often exhibit apraxia; a difficulty in performing skilled actions (Mühlau et al. 2005; Rounis et al. 2021). This suggests that the neural mechanism underlying motor imitation is predominantly left lateralized.

While our findings offer important insight into left hemisphere visual connectivity patterns in ASD, we must exercise caution in generalizing these results to the broader ASD population given the heterogeneous nature of ASD and that our ASD cohort was comprised of school‐age children, the majority of whom had at least normal range IQ.

Future studies should pursue longitudinal approaches to examine the developmental trajectories of HOV‐SM1 and HOV‐PFC connectivity from early childhood to adulthood in ASD and TD children as well as hemispheric differences in these trajectories. This could help us better understand how HOV connectivity profiles change with praxis outcomes over the course of development. Another area of interest is examining HOV connectivity patterns in the context of sensory preferences (e.g., proprioceptive over visual bias), which may provide further insight into the neural mechanisms underlying praxis. Additionally, a follow‐up study would be necessary to confirm if HOV‐PFC connectivity in ASD children is associated with measures of cognitive control and executive function. Findings that children with ASD rely more on top‐down processes in tasks requiring dynamic VMI could inform future interventions to improve learning of social and motor skills through observation and imitation.

## Conflicts of Interest

The authors declare no conflicts of interest.

## Supporting information


**Data S1:** aur70146‐sup‐0001‐Supinfo.docx.

## Data Availability

The data that supports the paper's findings is not publicly available due to ethical restrictions. Data can be made available upon reasonable request from Deana Crocetti at the Center for Neurodevelopmental and Imaging Research (CNIR) at Kennedy Krieger Institute.
