# Supplementary material for: Anomalous Pattern of Left Hemisphere Visual Connectivity in Children With Autism: Association With Impaired Praxis
Source: Autism Res. 2025 Nov 29;19(2):e70146. doi: 10.1002/aur.70146 (PMC12948742; doi:10.1002/aur.70146)
Supplement: Supplementary file 1 — Data S1: aur70146‐sup‐0001‐Supinfo.docx. [file AUR-19-0-s001.docx]

**Supplement**

1. **Diagnosis-by-ROI Associations with HOV Connectivity**

***Diagnosis-by-ROI Linear Mixed Effect Model***

$$FC \sim\beta_{0}+\beta_{1}\left( Diagnosis \right)+\beta_{2}\left( ROIs \right)+\beta_{3}\left( Diagnosis\times ROI \right)+ \beta_{4}\left( Age \right)+\beta_{5}\left( Sex \right)+\beta_{6}\left( Handedness \right)+\beta_{7}\left( Head Coil \right)+\beta_{8}\left( IQ \right)+u_{k}+\epsilon$$

Where $u_{k}$ represents the random effect term for subject ID number and $\epsilon$ the residual error term. The model also includes age, sex, handedness, head coil, and IQ as covariates.

**Table 4.** Both hemispheres linear mix effect model output. *p<0.01, **p<0.001

| Both Hemispheres | $\boldsymbol{\beta}$ | SE | df | t | p |
| --- | --- | --- | --- | --- | --- |
| (Intercept) | 0.387 | 0.117 | 420.6 | 3.299 | 0.001* |
| Diagnosis (ASD) | 0.019 | 0.024 | 562.1 | 0.812 | 0.417 |
| ROIs (SM1) | -0.019 | 0.01 | 425 | -1.94 | 0.054 |
| Age | -0.009 | 0.008 | 419 | -1.247 | 0.213 |
| Sex (Male) | 0.017 | 0.021 | 419 | 0.802 | 0.423 |
| Handedness (Mixed) | 0.139 | 0.05 | 419 | 0.281 | 0.779 |
| Handedness (Right) | 0.005 | 0.037 | 419 | 0.124 | 0.901 |
| Head Coil (8ch) | -0.017 | 0.019 | 419 | -0.886 | 0.376 |
| IQ | 0.001 | 0.001 | 419 | 1.125 | 0.261 |
| Diagnosis (ASD) x ROIs (SM1) | -0.038 | 0.019 | 425 | -2.034 | 0.043* |

**Table 5.** Left hemisphere linear mix effect model output. *p<0.01, **p<0.001

| Left Hemisphere | $\boldsymbol{\beta}$ | SE | df | t | P |
| --- | --- | --- | --- | --- | --- |
| (Intercept) | 0.377 | 0.123 | 420.9 | 3.061 | 0.002* |
| Diagnosis (ASD) | 0.023 | 0.026 | 592.9 | 0.898 | 0.37 |
| ROIs (SM1) | -0.035 | 0.012 | 425 | -0.294 | 0.769 |
| Age | -0.008 | 0.008 | 419 | -0.974 | 0.331 |
| Sex (Male) | 0.018 | 0.023 | 419 | 0.818 | 0.414 |
| Handedness (Mixed) | -0.002 | 0.052 | 419 | -0.045 | 0.964 |
| Handedness (Right) | -0.007 | 0.039 | 419 | -0.019 | 0.985 |
| Head Coil (8ch) | 0.014 | 0.02 | 419 | -0.687 | 0.493 |
| IQ | 0.001 | 0.001 | 419 | 0.91 | 0.364 |
| Diagnosis (ASD) x ROIs (SM1) | -0.057 | 0.022 | 425 | -2.636 | 0.009* |

**Table 6.** Right hemisphere linear mix effect model output. *p<0.05, **p<0.01, ***p<0.001

| Right Hemisphere | $\boldsymbol{\beta}$ | SE | df | t | p |
| --- | --- | --- | --- | --- | --- |
| (Intercept) | 0.414 | 0.118 | 421 | 3.499 | < .001*** |
| Diagnosis (ASD) | 0.014 | 0.025 | 595.1 | 0.551 | 0.582 |
| ROIs (SM1) | -0.055 | 0.011 | 425 | -4.81 | < .001*** |
| Age | -0.01 | 0.008 | 419 | -1.27 | 0.205 |
| Sex (Male) | 0.02 | 0.022 | 419 | 0.928 | 0.354 |
| Handedness (Mixed) | 0.025 | 0.05 | 419 | 0.497 | 0.62 |
| Handedness (Right) | 0.006 | 0.037 | 419 | 0.167 | 0.867 |
| Head Coil (8ch) | -0.02 | 0.02 | 419 | -1.027 | 0.305 |
| IQ | 0.001 | 0.001 | 419 | 1.146 | 0.252 |
| Diagnosis (ASD) x ROIs (SM1) | -0.02 | 0.021 | 425 | -0.976 | 0.33 |

1. **Diagnosis-by-HOV-SM1 Connectivity Associations with Praxis**

***Praxis Linear Mixed Effect Model (HOV-SM1 FC)***

$Praxis Average Total Errors \sim\beta_{0}+\beta_{1}\left( Diagnosis\times FC_{HOV\text{-}SM1} \right)+\beta_{2}\left( Age \right)+\beta_{3}\left( Sex \right)+\beta_{4}\left( Handedness \right)+\beta_{5}\left( Head Coil \right)+\beta_{6}\left( IQ \right)+\epsilon$

Where $\epsilon$ represents the residual error term. The model also includes age, sex, handedness, head coil, and IQ as covariates.

**Table 7.** Both hemispheres praxis average total errors and HOV-SM1 FC linear mix effect model output. *p<0.05, **p<0.01, ***p<0.001

| Both Hemispheres | $\boldsymbol{\beta}$ | SE | t | p |
| --- | --- | --- | --- | --- |
| (Intercept) | 70.83 | 8.17 | 8.67 | <.001*** |
| Diagnosis (ASD) | 26.28 | 2.86 | 9.18 | <.001*** |
| FC_HOV-SM1_ | 5.99 | 3.62 | 1.66 | 0.099 |
| Age | -3.01 | 0.54 | -5.57 | <.001*** |
| Sex (Male) | 2.88 | 1.56 | 1.85 | 0.065 |
| Handedness (Mixed) | -3.54 | 3.54 | -1 | 0.318 |
| Handedness (Right) | -0.6 | 2.77 | -0.22 | 0.829 |
| Head Coil (8ch) | 7.97 | 1.43 | 5.57 | <.001*** |
| IQ | -0.24 | 0.05 | -4.9 | <.001*** |
| Diagnosis (ASD) x FC_HOV-SM1_ | -21.44 | 6.88 | -3.12 | 0.002** |

| Model Fit Statistics | values | p |
| --- | --- | --- |
| R^2^ | 0.50 |  |
| Adjusted R^2^ | 0.49 |  |
| F(9,283) | 31.59 | <.001*** |

**Table 8.** Left hemisphere praxis average total errors and HOV-SM1 FC linear mix effect model output. *p<0.05, **p<0.01, ***p<0.001

| Left Hemisphere | $\boldsymbol{\beta}$ | SE | t | p |
| --- | --- | --- | --- | --- |
| (Intercept) | 69.67 | 8.1 | 8.6 | < .001*** |
| Diagnosis (ASD) | 27.55 | 2.67 | 10.33 | < .001*** |
| FC_HOV-SM1_ | 6.64 | 3.25 | 2.04 | 0.042* |
| Age | -3.02 | 0.53 | -5.66 | < .001*** |
| Sex (Male) | 2.98 | 1.54 | 1.94 | 0.054 |
| Handedness (Mixed) | -3.39 | 3.51 | -0.96 | 0.336 |
| Handedness (Right) | -0.42 | 2.74 | -0.15 | 0.878 |
| Head Coil (8ch) | 8.16 | 1.42 | 5.76 | < .001*** |
| IQ | -0.24 | 0.05 | -4.86 | < .001*** |
| Diagnosis (ASD) x FC_HOV-SM1_ | -25.76 | 6.43 | -4.01 | < .001*** |

| Model Fit Statistics | values | p |
| --- | --- | --- |
| R^2^ | 0.512 |  |
| Adjusted R^2^ | 0.496 |  |
| F(9,283) | 32.96 | < .001*** |

**Table 9.** Right hemisphere praxis average total errors and HOV-SM1 FC linear mix effect model output. *p<0.05, **p<0.01, ***p<0.001

| Right Hemisphere | $\boldsymbol{\beta}$ | SE | t | p |
| --- | --- | --- | --- | --- |
| (Intercept) | 72.18 | 8.2 | 8.8 | < .001*** |
| Diagnosis (ASD) | 23.9 | 2.82 | 8.49 | < .001*** |
| FC_HOV-SM1_ | 3.7 | 3.53 | 1.05 | 0.296 |
| Age | -3 | 0.54 | -5.51 | < .001*** |
| Sex (Male) | 2.82 | 1.57 | 1.8 | 0.073 |
| Handedness (Mixed) | -3.79 | 3.56 | -1.06 | 0.289 |
| Handedness (Right) | -0.83 | 2.78 | -0.3 | 0.766 |
| Head Coil (8ch) | 7.76 | 1.44 | 5.39 | < .001*** |
| IQ | -0.24 | 0.05 | -4.88 | < .001*** |
| Diagnosis (ASD) x FC_HOV-SM1_ | -14.42 | 6.57 | -2.2 | 0.029* |

| Model Fit Statistics | values | p |
| --- | --- | --- |
| R^2^ | 0.493 |  |
| Adjusted R^2^ | 0.477 |  |
| F(9,283) | 30.54 | < .001*** |

1. **Diagnosis-by-HOV-PFC Connectivity Associations with HOV Connectivity**

***HOV-PFC FC(z) vs. Praxis Average Total Errors***


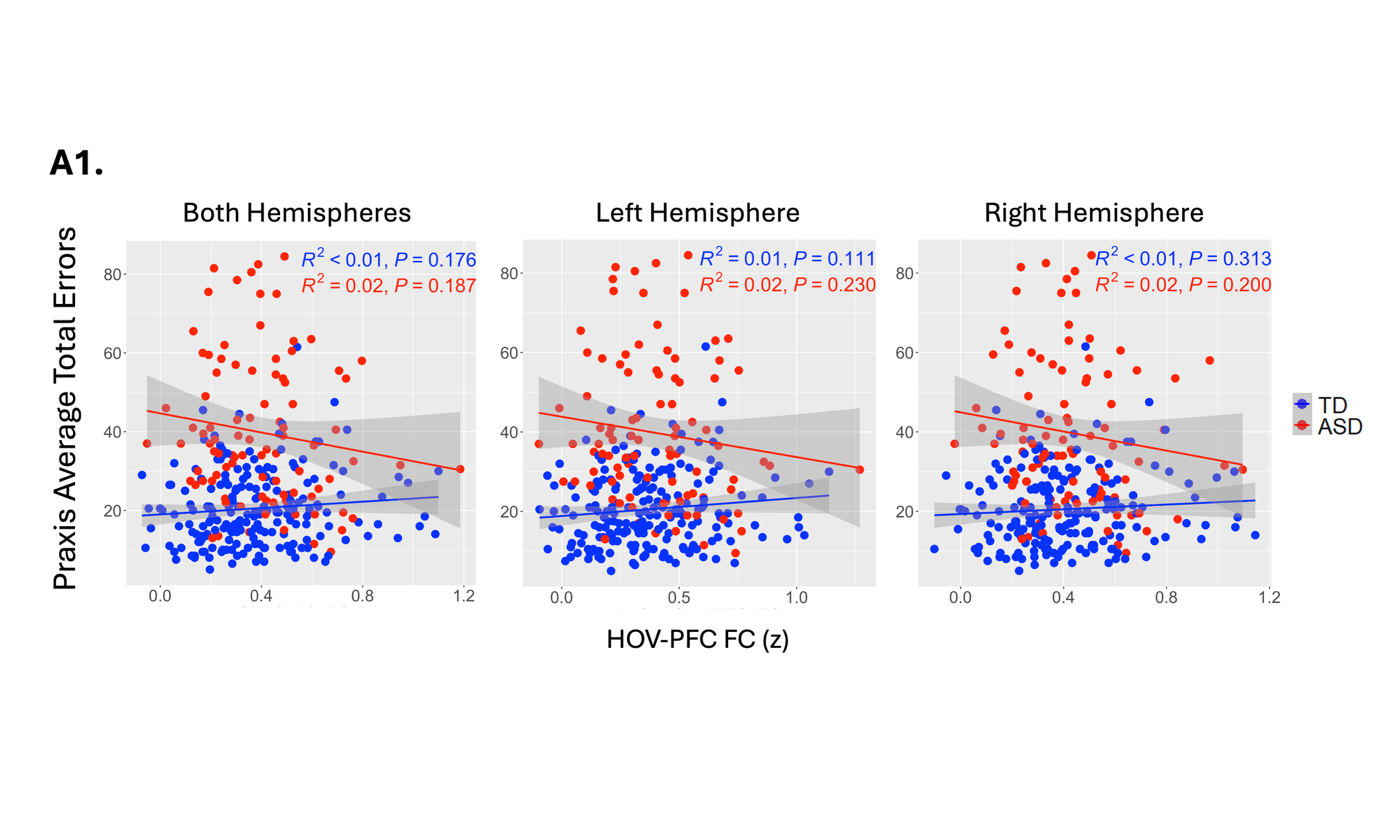


**Figure 3.** raw praxis average total errors plotted against HOV-SM1 FC (z) across hemispheres.

***Praxis Linear Mixed Effect Model (HOV-PFC FC)***

$Praxis Average Total Errors \sim\beta_{0}+\beta_{1}\left( Diagnosis\times FC_{HOV\text{-}PFC} \right)+\beta_{2}\left( Age \right)+\beta_{3}\left( Sex \right)+\beta_{4}\left( Handedness \right)+\beta_{5}\left( Head Coil \right)+\beta_{6}\left( IQ \right)+\epsilon$

Where $\epsilon$ represents the residual error term. The model also includes age, sex, handedness, head coil, and IQ as covariates.

**Table 10.** Both hemispheres praxis average total errors and HOV-PFC FC linear mix effect model output. *p<0.05, **p<0.01, ***p<0.001

| Both Hemispheres | $\boldsymbol{\beta}$ | SE | t | p |
| --- | --- | --- | --- | --- |
| (Intercept) | 71.46 | 8.22 | 8.69 | < .001*** |
| Diagnosis (ASD) | 25.74 | 3.05 | 8.44 | < .001*** |
| FC_HOV-PFC_ | 5.55 | 3.67 | 1.51 | 0.131 |
| Age | -2.99 | 0.54 | -5.48 | < .001*** |
| Sex (Male) | 2.77 | 1.57 | 1.77 | 0.078 |
| Handedness (Mixed) | -3.65 | 3.55 | -1.03 | 0.305 |
| Handedness (Right) | -0.6 | 2.77 | -0.22 | 0.829 |
| Head Coil (8ch) | 7.68 | 1.43 | 5.36 | < .001*** |
| IQ | -0.25 | 0.05 | -4.95 | < .001*** |
| Diagnosis (ASD) x FC_HOV-PFC_ | -17.9 | 6.72 | -2.66 | 0.008** |

| Model Fit Statistics | values | p |
| --- | --- | --- |
| R^2^ | 0.497 |  |
| Adjusted R^2^ | 0.481 |  |
| F(9,283) | 31.03 | < .001*** |

**Table 11.** Left hemisphere praxis average total errors and HOV-PFC FC linear mix effect model output. *p<0.05, **p<0.01, ***p<0.001

| Left Hemisphere | $\boldsymbol{\beta}$ | SE | t | p |
| --- | --- | --- | --- | --- |
| (Intercept) | 71.11 | 8.2 | 8.67 | < .001*** |
| Diagnosis (ASD) | 24.98 | 2.88 | 8.69 | < .001*** |
| FC_HOV-PFC_ | 6.36 | 3.51 | 1.81 | 0.071 |
| Age | -2.98 | 0.54 | -5.48 | < .001*** |
| Sex (Male) | 2.75 | 1.57 | 1.76 | 0.08 |
| Handedness (Mixed) | -3.57 | 3.55 | -1 | 0.317 |
| Handedness (Right) | -0.62 | 2.78 | -0.22 | 0.824 |
| Head Coil (8ch) | 7.74 | 1.43 | 5.4 | < .001*** |
| IQ | -0.25 | 0.05 | -4.93 | < .001*** |
| Diagnosis (ASD) x FC_HOV-PFC_ | -16.21 | 6.24 | -2.6 | 0.01** |

| Model Fit Statistics | values | p |
| --- | --- | --- |
| R^2^ | 0.496 |  |
| Adjusted R^2^ | 0.480 |  |
| F(9,283) | 31 | < .001*** |

**Table 12.** Right hemisphere praxis average total errors and HOV-PFC FC linear mix effect model output. *p<0.05, **p<0.01, ***p<0.001

| Right Hemisphere | $\boldsymbol{\beta}$ | SE | t | p |
| --- | --- | --- | --- | --- |
| (Intercept) | 71.87 | 8.28 | 8.67 | < .001*** |
| Diagnosis (ASD) | 25.39 | 3.23 | 7.87 | < .001*** |
| FC_HOV-PFC_ | 3.86 | 3.64 | 1.06 | 0.291 |
| Age | -2.98 | 0.55 | -5.46 | < .001*** |
| Sex (Male) | 2.84 | 1.57 | 1.81 | 0.072 |
| Handedness (Mixed) | -3.72 | 3.56 | -1.05 | 0.297 |
| Handedness (Right) | -0.65 | 2.79 | -0.23 | 0.816 |
| Head Coil (8ch) | 7.6 | 1.44 | 5.29 | < .001*** |
| IQ | -0.25 | 0.05 | -4.92 | < .001*** |
| Diagnosis (ASD) x FC_HOV-PFC_ | -15.96 | 6.8 | -2.35 | 0.02* |

| Model Fit Statistics | values | p |
| --- | --- | --- |
| R^2^ | 0.494 |  |
| Adjusted R^2^ | 0.478 |  |
| F(9,283) | 30.7 | < .001*** |

1. **Left Hemisphere HOV Connectivity Associations with Core Autism Symptoms (ADOS/ SRS)**

***FC(z) vs. ADOS Total Score***

***
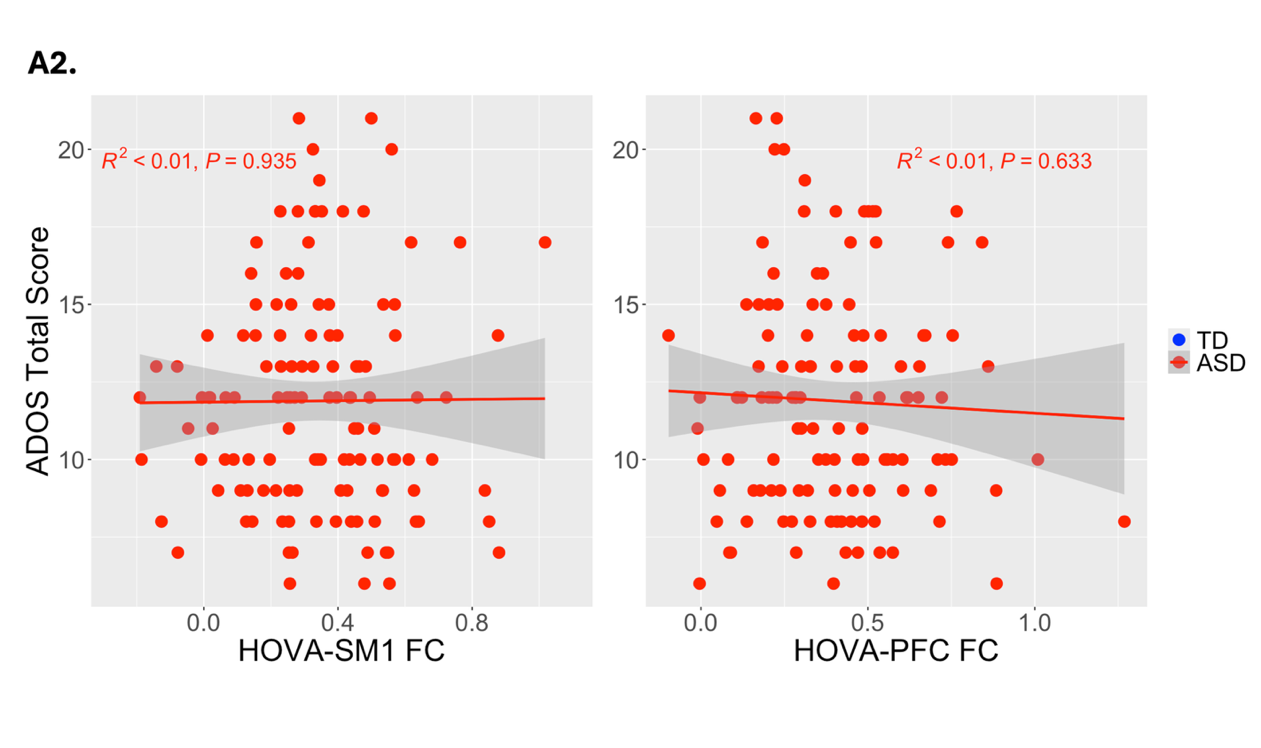
***

**Figure 4.** raw ADOS total score plotted against HOV-SM1 FC (z) (left) and HOV-PFC FC (z) (right) in left hemispheres.

***FC(z) vs. SRS Total Score***

***
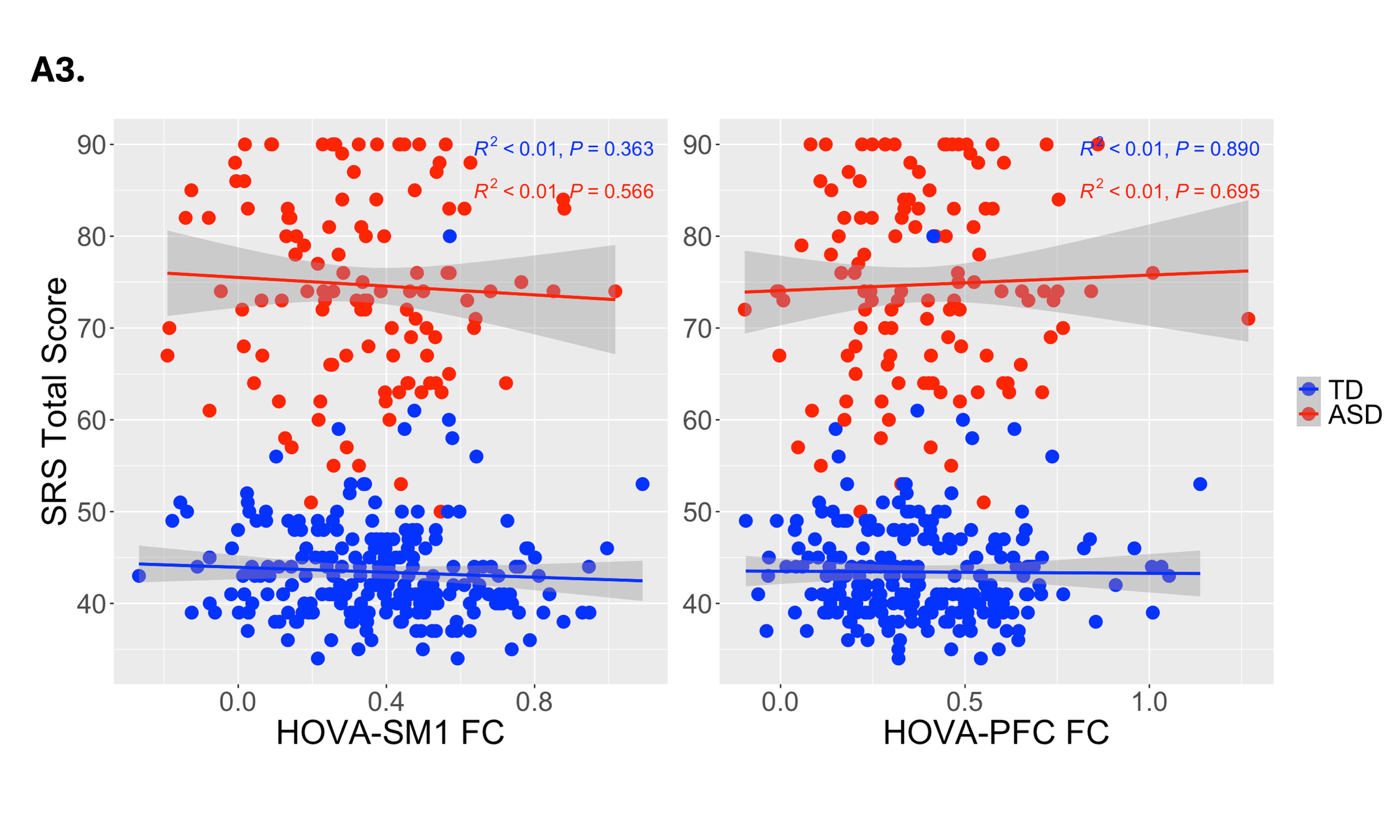
***

**Figure 5.** raw SRS total score plotted against HOV-SM1 FC (z) (left) and HOV-PFC FC (z) (right) in left hemispheres.

1. **ASD Sample Characterization**

***Diagnostic Criteria***

Children were excluded if they had a) a history of a definitive neurological disorder, including seizures (except for uncomplicated brief febrile seizures), tumor, lesion, severe head injury, or stroke, based on parent responses during an initial phone screening; b) a major visual impairment; or c) conditions that contraindicate or make it challenging to obtain MRI data (e.g., cardiac pacemaker, surgical clips in the brain or blood vessels, or dental braces). Children with other psychiatric diagnosis including anxiety disorders other than OCD, were included in our sample.

A diagnosis of ASD was determined using the Autism Diagnostic Observation Schedule-Generic (Lord et al., 2000) or the Autism Diagnostic Observation Schedule, Second Edition (ADOS-2, Lord and Jones, 2012), depending on the date of enrollment. Diagnosis was verified by a board-certified child neurologist (SHM) with more than 30 years of experience in the clinical assessment of autistic children. Autistic children were excluded if they had identifiable causes of autism (e.g., fragile X syndrome, Tuberous Sclerosis, phenylketonuria, congenital rubella), documented history of prenatal/perinatal insult, or showed evidence of meeting criteria for major depression, bipolar disorder, conduct disorder, or adjustment disorder based on parent responses during an initial phone screening.

Within the ASD group, a secondary diagnosis of attention deficit hyperactivity disorder (ADHD) was determined using the DSM-IV or DSM5 (American Psychiatric Association, 2000, 2013) criteria and confirmed using a structured parent interview, either the Diagnostic Interview for Children and Adolescents-IV (DICA-IV, Reich, 2000) or the Kiddie Schedule for Affective Disorders and Schizophrenia for School-Age Children (K-SADS, Kaufman et al., 2013), as well as parent and teachers versions of the Conners-Revised (Conners, 1999) or the Conners-3 Rating Scale (Conners, 2008), and parent and teacher versions of the DuPaul ADHD Rating Scale (DuPaul et al., 1998). To be classified as having comorbid ASD and ADHD (ASD+ADHD), a child with ASD had to receive one of the following: 1) a t-score of 60 or higher on the inattentive or hyperactive subscales of the Conners Parent or Teacher Rating Scale, or 2) a score of 2 or 3 on at least 6 of 9 items on the Inattentive or Hyperactivity/Impulsivity scales of the ADHD Rating Scale-IV (DuPaul et al., 1998). Diagnosis was verified by a board-certified child neurologist (SHM) or clinical psychologist with extensive experience in the clinical assessment of children with ADHD. Children taking stimulant medications were asked to withhold their medications the day prior to and the day of their study visit to avoid the effects of stimulants on

cognitive, behavioral, and motor measures.

Children were excluded from the typically developing group if they had a first-degree relative with ASD, if parent responses to either the DICA-IV or for more recent participants, the K-SADS, revealed a history of a developmental or psychiatric disorder, except for simple phobias, or if they scored above clinical cut-offs on the parent and teacher versions of the Conners and ADHD Rating Scales.

**Table 13.** ASD sample (n=127) comorbidities

| ASD Comorbidities | *Prevalence* | *N* |
| --- | --- | --- |
| ADHD | 60.63% | 77 |
| Anxiety Disorder | 26.77% | 34 |
| Mood Disorder | 1.57% | 2 |
| Trauma | 0.80% | 1 |
| Disruptive Disorder | 13.39% | 17 |
| OCD | 7.09% | 9 |

**References**

American Psychiatric Association (2000). Diagnostic and Statistical Manual of Mental Disorders. (DSM-IV®) (4th ed), American Psychiatric Association (2000)

American Psychiatric Association (2013). Diagnostic and Statistical Manual of Mental Disorders. (DSM-5®) (5th ed.), American Psychiatric Association (2013), 10.1176/appi.books.9780890425596

Conners, C. K. (1999). Conners Rating Scales-Revised. In M. E. Maruish (Ed.), The use of psychological testing for treatment planning and outcomes assessment (2nd ed., pp. 467–495). Lawrence Erlbaum Associates Publishers.

Conners, C. K., Pitkanen, J., & Rzepa, S. R. (2011). Conners 3rd Edition (Conners 3; Conners 2008). In J. S. Kreutzer, J. DeLuca, & B. Caplan (Eds.), Encyclopedia of Clinical Neuropsychology (pp. 675–678). Springer New York. <https://doi.org/10.1007/978-0-387-79948-3_1534>

DuPaul, G. J., Power, T. J., Anastopoulos, A. D., & Reid, R. (1998). ADHD Rating Scale—IV: Checklists, norms, and clinical interpretation. The Guilford Press.

Kaufman, J., Birmaher, B., Brent, D., Rao, U., Flynn, C., Moreci, P., Williamson, D., & Ryan, N. (1997). Schedule for affective disorders and schizophrenia for school-age children-present and lifetime version (K-sads-pl): Initial reliability and validity data. Journal of the American Academy of Child & Adolescent Psychiatry, 36(7), 980–988. <https://doi.org/10.1097/00004583-199707000-00021>

Lord, C., & Jones, R. M. (2012). Annual Research Review: Re‐thinking the classification of autism spectrum disorders. Journal of Child Psychology and Psychiatry, 53(5), 490–509. <https://doi.org/10.1111/j.1469-7610.2012.02547.x>

Lord, C., Risi, S., Lambrecht, L., Cook, E. H., Leventhal, B. L., DiLavore, P. C., Pickles, A., & Rutter, M. (2000). The autism diagnostic observation schedule-generic: A standard measure of social and communication deficits associated with the spectrum of autism. Journal of Autism and Developmental Disorders, 30(3), 205–223.

Reich, W. (2000). Diagnostic interview for children and adolescents(Dica). Journal of the American Academy of Child & Adolescent Psychiatry, 39(1), 59–66. <https://doi.org/10.1097/00004583-200001000-00017>
